# Effects of Wannachawee Recipe with Antipsoriatic Activity on Suppressing Inflammatory Cytokine Production in HaCaT Human Keratinocytes

**DOI:** 10.1155/2017/5906539

**Published:** 2017-08-16

**Authors:** Mingkwan Na Takuathung, Ariyaphong Wongnoppavich, Pornsiri Pitchakarn, Ampai Panthong, Parirat Khonsung, Natthakarn Chiranthanut, Noppamas Soonthornchareonnon, Seewaboon Sireeratawong

**Affiliations:** ^1^Department of Pharmacology, Doctor of Philosophy Program in Pharmacology, Faculty of Medicine and Graduate School, Chiang Mai University, Muang 50200, Thailand; ^2^Department of Pharmacology, Faculty of Medicine, Chiang Mai University, Muang, Chiang Mai 50200, Thailand; ^3^Department of Biochemistry, Faculty of Medicine, Chiang Mai University, Muang, Chiang Mai 50200, Thailand; ^4^Department of Pharmacognosy, Faculty of Pharmacy, Mahidol University, Rajathevi, Bangkok 10400, Thailand

## Abstract

Psoriasis is a chronic inflammatory and immune-mediated skin disease. The pathogenesis involves T cells activation via the IL-23/Th17 axis. Conventional treatments of psoriasis have adverse events influencing patients' adherence. Wannachawee Recipe (WCR) has been effectively used as Thai folk remedy for psoriasis patients; however, preclinical evidence defining how WCR works is still lacking. This study defined mechanisms for its antiproliferation and anti-inflammatory effects in HaCaT cells. The cytotoxicity and antiproliferation results from SRB and CCK-8 assays showed that WCR inhibited the growth and viability of HaCaT cells in a concentration-dependent manner. The distribution of cell cycle phases determined by flow cytometry showed that WCR did not interrupt cell cycle progression. Interestingly, RT-qPCR revealed that WCR significantly decreased the mRNA expression of IL-1*β*, IL-6, IL-8, IL-17A, IL-22, IL-23, and TNF-*α* but induced IL-10 expression in TNF-*α*- and IFN-*γ*-induced HaCaT cells. At the protein level determined by ELISA, WCR significantly reduced the secretion of IL-17A, IL-22, and IL-23. The WCR at low concentrations was proved to possess anti-inflammatory effect without cytotoxicity and it did not interfere with cell cycle of keratinocytes. This is the first study to provide convincing evidence that WCR is a potential candidate for development of effective psoriasis therapies.

## 1. Introduction

Psoriasis is a chronic immune-mediated skin disease manifesting as focal formation of inflamed and scaly plaques derived from excessive proliferation of keratinocytes [1]. Interleukin 23 (IL-23) and interleukin 17 (IL-17), which are cytokines belonging to a subset of T-helper 17 (Th 17), play a major role in pathogenesis of psoriasis [2]. IL-23 functions as a key cytokine for stimulating survival and expansion of Th17 cells [3]. After this stimulation, the downstream effector molecules of Th17 cells, including IL-17A, IL-22, TNF-*α*, and IL-6 are produced in response to certain stimuli [2, 4] and subsequently induce keratinocyte proliferation and other hallmark features of psoriasis [5].

Choice of psoriatic treatment depends on many factors such as severity of the disease and its impact on patient's life [6]. For mild psoriasis, corticosteroids, vitamin D3 analogues, and calcineurin inhibitors have been currently used as topical therapies. For severe psoriasis, phototherapy or conventional systemic agents including methotrexate, cyclosporine, acitretin, and, in some countries, fumaric acid esters are chosen depending on the patient's condition [7]. Recently, biological therapies, which are proteins or antibodies targeting specific inflammatory mediators and T cells, have been developed and approved for effective psoriasis treatment [8]. However, as a great concern about these therapies, the effect of long-term chronic immunosuppression has potential to increase the risks of infection and cancer [6]. In addition, the well-documented arrays of adverse effects result in decrease of patient's adherence. Therefore, there is a need for novel antipsoriatic drug that would provide improved effectiveness but with fewer side effects and lower costs. One of possible solutions is traditional medicine because it provides front-line pharmacotherapy for billions of people worldwide. However, its efficacy related to sufficient preclinical and clinical evidence is often skeptical by the Western medical establishment [9].

Thai traditional medicine employs many different plants for the folk remedy of dermatological conditions [10]. Wannachawee Recipe (WCR) has been enlisted in the Hospital Traditional Medicine Formulary and has been used for the treatment of psoriasis in the Thai Traditional Medicine Clinic of Prapokklao Hospital since 2006. An observational study (2006–2011) indicates that psoriasis accounted for the highest amount of patient attendance at this clinic. There were psoriasis patients (between 13 and 97 years old) with different severity of psoriasis (severe 16.9%, moderate 45.6%, and mild 37.5%). For the treatment regimen, patients were prescribed to take 10.77 g of WCR per day for 6-7 months, and the severity of psoriasis was determined again by certified dermatologists. The results revealed that 93% of 136 Thai psoriasis patients had good response with WCR, whereas 7% of them showed less improvement. The factors associated with aggravation of psoriasis among these patients were suggested to be related to particular diet, sleep deprivation, stress, and ultraviolet light and chemical exposure [11]. Although the WCR has been successfully used as a clinical therapy at the Thai Traditional Medicine Clinic for many years, there is still a lack of supporting preclinical evidence supporting the mechanism of pharmacological action of the WCR in psoriasis treatment. The present study aimed to investigate specific mechanism underlying the therapeutic potential of WCR on antiproliferation and anti-inflammatory effects in HaCaT human keratinocytes.

## 2. Materials and Methods

### 2.1. Chemicals and Reagents

Unless stated, all chemicals and reagents were purchased from Sigma Aldrich Co. (Merck KGaA, Darmstadt, Germany,). Acitretin 25 mg was obtained from Silom Medical Co., Ltd. (Thailand), and dimethyl sulfoxide (DMSO) from AMRESCO LLC (OH, USA).

### 2.2. Cell Line

An immortalized human epidermal keratinocyte cell line, HaCaT cell, was purchased from Cell Lines Service GmbH (Eppelheim, Baden-Württemberg, Germany). They were cultured in Dulbecco's modified Eagle's medium (DMEM/high glucose, Gibco Inc., Grand Island, NY), supplemented with 10% heat-inactivated fetal bovine serum (FBS) and antibiotics (100 U/mL penicillin and 100 *μ*g/mL streptomycin) (all from Gibco Inc.) and maintained at 37°C in a humidified atmosphere at 5% CO_2_.

### 2.3. Plant Materials

WCR is composed of 8 Thai herbs as shown in Table 1. Roots of* S. involuta* and* S. collinsae* were purchased from Chachoengsao Province, Thailand, whereas the other seven herbal plants were from Chanthaburi contract farming of Thai Traditional Medicine Clinic of Prapokklao Hospital. All herbal plants were primarily verified by qualified Thai traditional doctors. The qualitative and quantitative analyses for both herbal plants and WCR were performed according to the methods in Thai Herbal Pharmacopoeias and WHO guideline [12, 13], by Assoc. Professor Dr. Noppamas Soonthornchareonnon, Mahidol University, Bangkok, Thailand.

Each crude herb was dried in a hot air oven (60°C) for 3 hours; then it was grounded and sieved. All crude components of WCR were mixed in distilled water and refluxed at 80–100°C for 1 hour. The first extract was percolated through a filter cloth, while fresh distilled water was added repeatedly to the crude residual. This second extract was percolated through a filter cloth. The mixtures of the first and second extracts were concentrated by a rotary evaporator under reduced pressure until the water had evaporated and dried by spray apparatus. The yield of WCR was 11.77% of the fresh weight. The final concentration of WCR crude extract (100 mg/mL) was prepared by dissolving in DMSO.

### 2.4. Sulforhodamine B (SRB) Assay

HaCaT cells were seeded in 96-well microtiter plates at a density of 10,000 cells/well and incubated at 37°C for 24 h. Control cells were cultured in fresh medium containing no drug. Acitretin and methotrexate were used as positive controls for further experiments; therefore the cytotoxic effects of these two drugs were determined. Cells were treated in pentaplicate with 8 concentrations of either methotrexate (0–25 *μ*M), acitretin (0–100 *μ*M), or WCR (0–1000 *μ*g/mL) and incubated for 24 h. SRB colorimetric assay was performed as previously described by Skehan et al. (1990) [14] and Vichai and Kirtikara (2006) [15]. Briefly, cells were fixed with 100 *μ*L of cold 10% (wt/vol) trichloroacetic acid (TCA) at 4°C for 1 h. The plates were washed and dried four times and then stained with 100 *μ*L of 0.057% (wt/vol) SRB dissolved in 1% acetic acid for 30 min. The plates were subsequently rinsed four times with 1% (vol/vol) acetic acid to remove unbound dye. The protein-bound dye was solubilized with 200 *μ*L of 10 mM Tris base solution (pH 10.5) and the absorbance at 510 nm was determined using the Synergy™ H4 Hybrid Multi-Mode microplate reader (BioTek Instruments, Inc., Vermont, USA). The percentage of growth inhibition was calculated using the equation shown below. Day 0 means the day that treatment was initiated and cells were fixed after treatment for a no-growth control.

(1) %  control  cell  growth=mean  ODsample−mean  ODday  0mean  ODneg  control−mean  ODday  0×100,%  growth  inhibition=100−%  of  control  cell  growth.

For IC_50_ determination, a dose-response curve between test compound concentrations and percent growth inhibition was plotted. IC_50_ values were derived using curve-fitting methods.

### 2.5. CCK-8 Assay

Cell viability of HaCaT cells was measured with Cell Counting Kit-8 assay (CCK-8 from Dojindo, Kumamoto, Japan) according to the manufacturer's instruction. HaCaT cells were seeded at a density of 10,000 cells/well in 96-well plates and incubated at 37°C for 24 h. Twenty-four hours after the treatment with methotrexate (0–25 *μ*M), acitretin (0–100 *μ*M), or WCR (0–1000 *μ*g/mL), 10 *μ*L of CCK-8 was added to each well of the 96-well plates and incubated for 3 h in a CO_2_ incubator. The absorbance of the reaction was measured at 450 nm with the Synergy H4 Hybrid Multi-Mode microplate reader (BioTek Instruments, Inc., Vermont, USA). The absorbance read from each well was calculated using the equation below to obtain the percentage of cell viability.

(2) %  cell  viability=mean  ODsample−mean  ODblankmean  ODneg  control−mean  ODblank×100.

### 2.6. Cell Cycle

HaCaT cells with a density of 3 × 10^5^ cells/well were cultured in 6-well plates for 24 h prior to the experiment. The cells were serum-starved for 24 h before the treatment. After 48 h of methotrexate (0.2 *μ*M), acitretin (20 *μ*M), or WCR (25, 50, and 75 *μ*g/mL) treatment, cells were harvested by trypsinization. The cells were washed twice with phosphate-buffered saline (PBS) prior to fixation with cold 70% ethanol. The fixed cells were refrigerated for at least 4 h at 4°C before being stained with Guava® Cell Cycle Reagent (Merck Millipore, CA, USA), which was a fluorogenic compound containing propidium iodide (PI). The stained cells were incubated for 30 min, shielded from the light, and measured for the distribution of the cell cycle phases by using Guava easyCyte Flow Cytometers (Merck Millipore, CA, USA). Data were analyzed using Guava Cytosoft™ version 4.2 software (Millipore) according to the manufacturer's instructions.

### 2.7. Real-Time Reverse Transcription Polymerase Chain Reaction (RT-qPCR)

HaCaT cells (3 × 10^5^ cells/well) were seeded into 6-well plates and incubated 24 h. The cells were pretreated with 20 *μ*M of acitretin or 50–100 *μ*g/mL of WCR for 1 h and inflammation was stimulated with the addition of TNF-*α* and IFN-*γ* (10 ng/mL each) (PEPROTECH, USA) for 12 h before harvesting cells.

To detect the mRNA expression, total RNA was extracted by using FavorPrep™ Total RNA Purification Mini Kit (Favorgen Biotech Corp., Taiwan). The quantity of total RNA was estimated using Nano-Drop spectrophotometer (Thermo Fisher Scientific, USA). RNA was reverse transcribed using ReverTra Ace® qPCR RT Master mix with gDNA Remover (TOYOBO, Japan), following the manufacturer's instruction. Six microliters of RNA template (1.5 *μ*g/*μ*L) was gently added to 6 *μ*L of 4x DN Master Mix (containing gDNA remover) and 12 *μ*L nuclease-free water, and the mixture was incubated for 5 min at 37°C. The solution was added with 6 *μ*L of 5x RT Master Mix II, and reverse transcription was performed (37°C for 15 min, 50°C for 5 min, and 98°C for 5 min). The resulting complementary (c)DNA was used as a template for quantitative PCR using the PCRmax Eco 48 real-time PCR system (PCRmax Limited, UK) with PerfeCTa™ SYBR® Green FastMix™, Low Rox™ (Quanta Biosciences™, USA), in accordance with the manufacturer's protocol. The reaction mixture consisted of 2 *μ*L cDNA, 1 *μ*L nuclease-free water, 5 *μ*L PerfeCTa SYBR Green FastMix, Low Rox, and 1 *μ*L (100 nM) of each specific primer (Table 2). PCR was performed under the following conditions: 95°C for 3 min, 45 cycles of 95°C for 10 s, 56°C for 15 s, 72°C for 30 s, 95°C for 15 s to detect the melting curve, 55°C for 15 s, and 95°C for 15 s.

### 2.8. Enzyme-Linked Immunosorbent Assay (ELISA)

TNF-*α* and IFN-*γ* can induce production and secretion of several inflammatory cytokines in HaCaT cells. The concentration of IL-17A, IL-22, and IL-23 in the supernatant of HaCaT cells was measured using human IL-17A, IL-22, and IL-23 ELISA MAX™ Deluxe (BioLegend, USA). ELISA was performed as recommended by the manufacturer. Briefly, the ELISA plate was coated with 100 *μ*L of 1x capture antibody in coating buffer and incubated overnight at 4°C. The plate was washed with wash buffer (0.05% Tween-20 in PBS) four times and then blocked with 200 *μ*L of blocking buffer for 1 h at room temperature. After blocking, the plate was washed with wash buffer for four times. Human IL-17A, IL-22, and IL-23 standards were diluted to reach the concentrations of 0–1000 pg/mL. One hundred microliters of the standards or samples was added to each well in triplicate for 2 h at room temperature. The ELISA plate was washed four times, added with 100 *μ*L of the specific detection antibody for 1 h at room temperature, and washed four times. Avidin-HRP conjugated (1 : 1000) (100 *μ*L) was added and incubated for 30 min followed by washing for five times. Finally, the freshly mixed TMB substrate solution (100 *μ*L) was added into each well and incubated in the dark for 30 min. The stop solution (2N H_2_SO_4_) (100 *μ*L) was added to each well and the absorbance at 450 nm and 570 nm was measured within 15 min by using the Synergy H4 Hybrid Multi-Mode microplate reader (BioTek Instruments, Inc., Vermont, USA).

### 2.9. Statistical Analysis

Data were analyzed by one-way analysis of variance (one-way ANOVA), followed by post hoc Fisher's least significant difference (LSD) test using the SPSS for Windows (version 20.0). Differences were considered significant at *p* < 0.05. All experiments were performed in triplicate using a minimum of three experiment trials. All values are expressed as mean ± standard error of mean (SEM).

## 3. Results

### 3.1. Cytotoxicity of the WCR on HaCaT Cells

**Figure 1:**
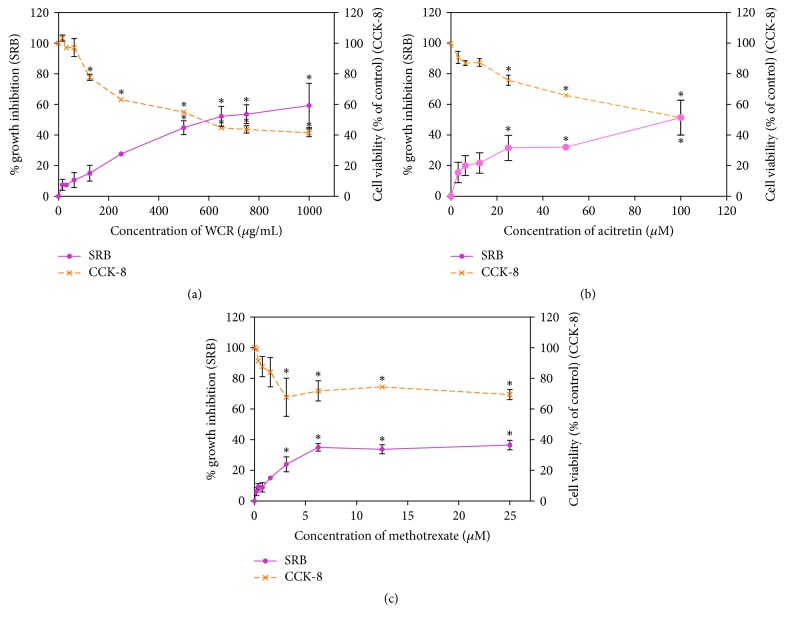
Growth inhibition and cell viability of HaCaT cells treated with WCR. (a) Cells treated with WCR (0–1000 *μ*g/mL), (b) cells treated with acitretin (0–100 *μ*M), and (c) cells treated with methotrexate (0–25 *μ*M). Data are presented as mean ± SEM (*n* = 4). ^*∗*^*p* < 0.05 compared with untreated cells.

To examine their cytotoxicity, HaCaT cells were treated with different concentrations of WCR, acitretin, and methotrexate for 24 h. The cell growth and viability were determined by SRB and CCK-8 assays, respectively. As shown in Figure 1(a), WCR inhibited the growth of keratinocytes in a concentration-dependent manner, with a maximum inhibition around 60% for the highest concentration tested (1000 *μ*g/mL). Likewise, CCK-8 assay showed that the cell viability was reduced in a concentration-dependent manner. The IC_50_ values from SRB and CCK-8 assays after the 24 h treatment of WCR were approximately 643.90 ± 73 *μ*g/mL and 575 ± 35.96 *μ*g/mL, respectively. Acitretin (Figure 1(b)) and methotrexate (Figure 1(c)) were used as a positive control which undoubtedly showed effective inhibition of cell growth and cell viability at low concentrations. The nontoxic concentrations of drugs and WCR were chosen to further evaluate changes in molecular events.

### 3.2. Flow Cytometric Analysis of HaCaT Cells

**Figure 2:**
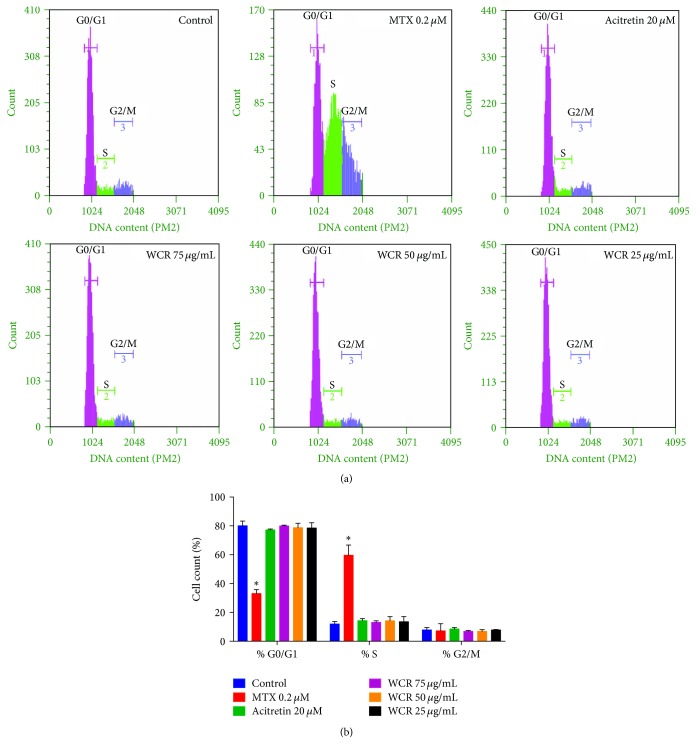
Percentage of cell cycle distribution (G0/G1, S, and G2/M phases) of cells treated with methotrexate, WCR, and acitretin. (a) Typical histogram from flow cytometry with PI staining. (b) Histogram representing cell cycle distribution of HaCaT cells treated with medium alone (control), 0.2 *μ*M of methotrexate (MTX), 20 *μ*M of acitretin, 75 *μ*g/mL of WCR, 50 *μ*g/mL of WCR, or 25 *μ*g/mL of WCR. Data are expressed as mean ± SEM (*n* = 3). ^*∗*^*p* < 0.0001 compared with control cells.

For better understanding the mechanism of inhibition of cell proliferation, possible cell arrest at specific phases of the cell cycle was analyzed following treatment of HaCaT with methotrexate, acitretin, or WCR for 48 h. Cell viability and cytotoxicity tests at 48 h were also performed and the trend was similar to that in cells treated with methotrexate, acitretin, or WCR for 24 h (data not shown). The amount of living cells was measured through stoichiometric detection of nucleic acid bound with PI and sent off fluorescent emission proportional to the DNA content of a cell [16]. The flow cytometric data on PI-stained HaCaT cells are shown in Figure 2. The treatment of WCR (25, 50, and 75 *μ*g/mL) and acitretin (20 *μ*M) at nontoxic concentrations had no effect on cell cycle distribution compared with the control group. However, methotrexate treatment significantly increased the percentage of cells at the S-phase.

### 3.3. Inhibitory Effects of WCR on mRNA Expression of Cytokines in TNF-*α*- and IFN-*γ*-Stimulated HaCaT Cells

**Figure 3:**
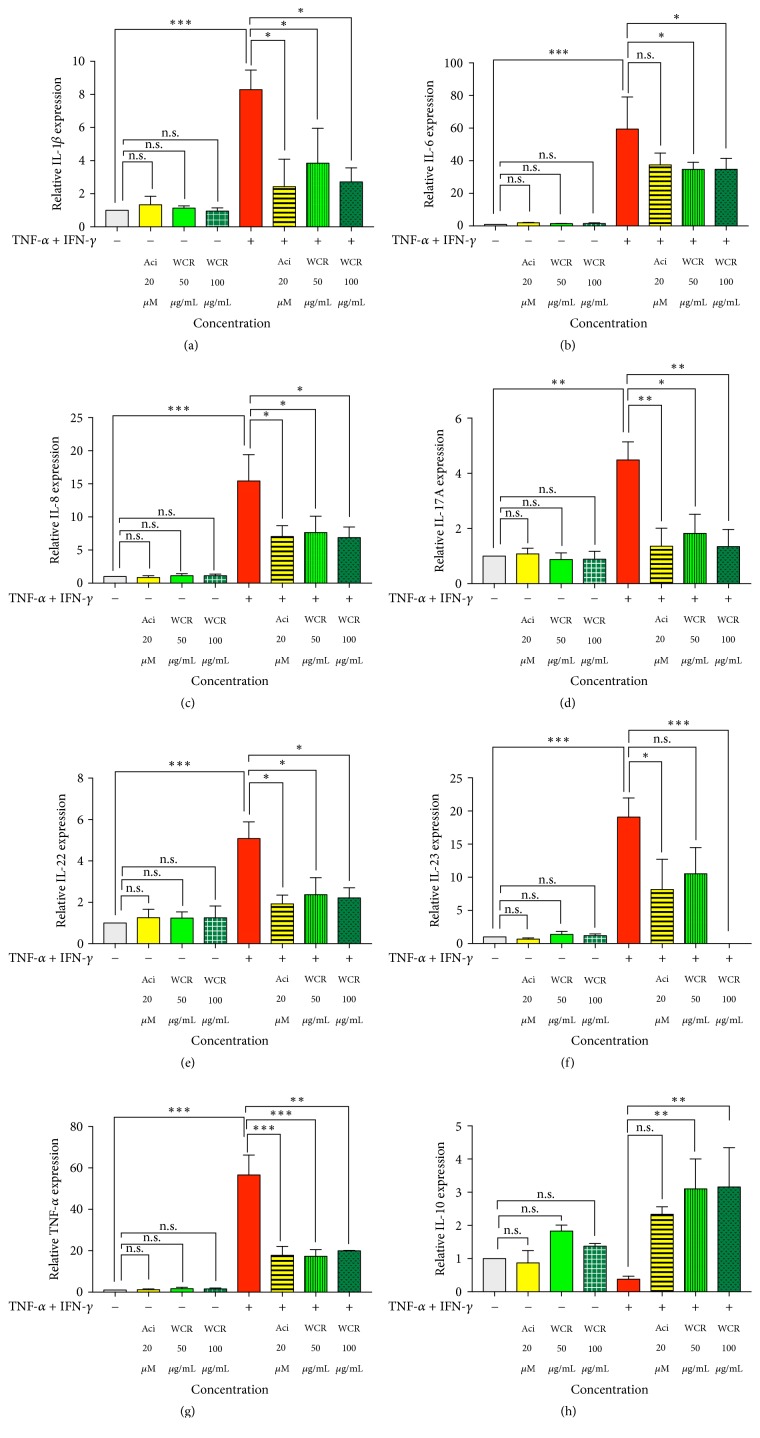
Effects of WCR on cytokine expression in TNF-*α*- and IFN-*γ*-stimulated HaCaT cells. Relative expression levels of IL-1*β* (a), IL-6 (b), IL-8 (c), IL-17A (d), IL-22 (e), IL-23 (f), TNF-*α* (g), and IL-10 (h). Data are expressed as mean ± SEM of three independent experiments; n.s., not significant. ^*∗*^*p* < 0.05, ^*∗∗*^*p* < 0.005, and ^*∗∗∗*^*p* < 0.001 compared with TNF-*α* and IFN-*γ* treated cells.

To investigate the effect of WCR on suppressing the production of proinflammatory cytokines in the keratinocytes, HaCaT cells were exposed to TNF-*α* (10 ng/mL) and IFN-*γ* (10 ng/mL) with or without the presence of WCR. As shown in Figure 3, the upregulation of IL-1*β*, IL-6, IL-8, IL-17A, IL-22, IL-23, and TNF-*α* expression was markedly decreased in the groups treated with acitretin and WCR. In contrast, the presence of acitretin and WCR significantly potentiated TNF-*α*- and IFN-*γ*-induced IL-10 gene expression (Figure 3(h)). Specifically, the inhibitory activities of 20 *μ*M acitretin, 50 *μ*g/mL WCR, and 100 *μ*g/mL WCR on IL-1*β*, IL-17A, and IL-22 expression in HaCaT cells were approximately 3-fold, 2-fold, and 3-fold, respectively, greater than the cells stimulated with TNF-*α* and IFN-*γ* alone (Figures 3(a), 3(d), and 3(e)). Moreover, in comparison to HaCaT cells with TNF-*α* and IFN-*γ* stimulation alone, approximately a 2-fold decrease in IL-6 and IL-8 expression and a 3-fold decrease in TNF-*α* expression were observed in the groups treated with both 20 *μ*M of acitretin and 50–100 *μ*g/mL of WCR (Figures 3(b), 3(c), and 3(g)). Furthermore, the presence of acitretin 20 *μ*M, WCR 50 *μ*g/mL, and WCR 100 *μ*g/mL suppressed the expression of IL-23 for 2-fold, 2-fold, and 19-fold, respectively, as compared with HaCaT cells stimulated with TNF-*α* and IFN-*γ* alone (Figure 3(f)). Interestingly, we discovered that the induction of HaCaT cells by TNF-*α* and IFN-*γ* decreased IL-10 expression three times lower than the negative control cells. However, after the treatment with 20 *μ*M of acitretin, 50 *μ*g/mL of WCR, and 100 *μ*g/mL of WCR, IL-10 expression was 2-fold, 3-fold, and 3-fold higher than cells treated with TNF-*α* and IFN-*γ* alone (Figure 3(h)).

### 3.4. Inhibitory Effects of WCR on Cytokine Secretion in TNF-*α*- and IFN-*γ*-Stimulated HaCaT Cells

**Figure 4:**
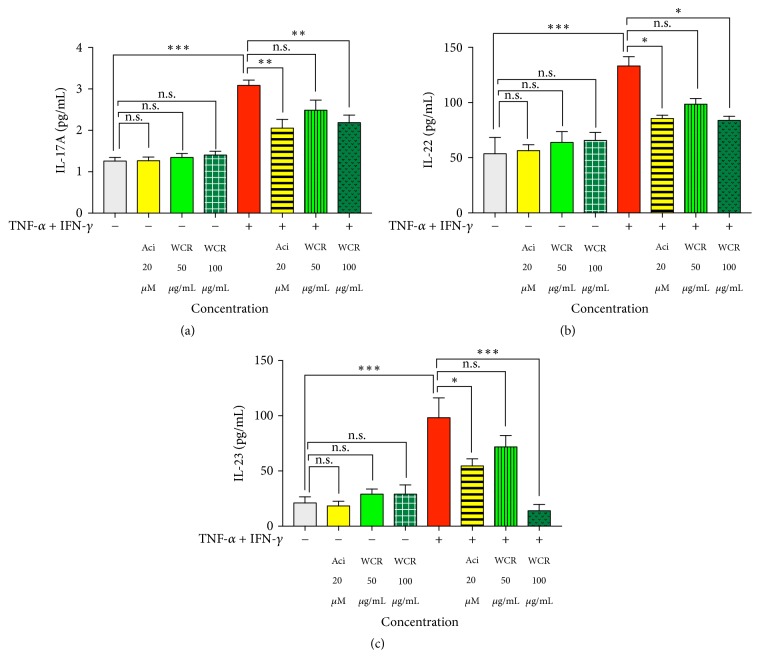
Effects of WCR on cytokine secretion in TNF-*α*- and IFN-*γ*-stimulated HaCaT cells. ELISA was performed to determine the concentration of IL-17A, IL-22, and IL-23 in the culture supernatants of cells treated with TNF-*α* and IFN-*γ* (each 10 ng/mL) for 24 h with or without the presence of WCR (50 and 100 *μ*g/mL). Acitretin (20 *μ*M) was used as a positive control; n.s., not significant. Data are expressed as mean ± SEM of three independent experiments. ^*∗*^*p* < 0.05, ^*∗∗*^*p* < 0.005, and ^*∗∗∗*^*p* < 0.001 compared with TNF-*α* and IFN-*γ* treated cells.

Focusing on three major proinflammatory cytokines (IL-17A, IL-22, and IL-23) responsible for development of psoriasis, the secretion of IL-17A, IL-22, and IL-23 in the culture supernatants of cells stimulated with TNF-*α* and IFN-*γ*, with or without the presence of WCR, was quantitatively evaluated by ELISA. As shown in Figure 4, cells stimulated with TNF-*α* and IFN-*γ* significantly increased the levels of IL-17A, IL-22, and IL-23 in the culture supernatants of HaCaT cells to 2-fold, 2-fold, and 3-fold, respectively, compared with the nonstimulated cells (*p* < 0.001). Although the production levels of IL-17A, IL-22, and IL-23 in the stimulated cells were not statistically different from the cells treated with the 50 *μ*g/mL WCR, treatment with 100 *μ*g/mL of WCR, and 20 *μ*M of acitretin significantly reduced the TNF-*α*- and IFN-*γ*-stimulated IL-17A and IL-22 production for 1.5-fold (*p* < 0.05) (Figures 4(a) and 4(b)). In addition, the presence of 20 *μ*M acitretin, 50 *μ*g/mL WCR, and 100 *μ*g/mL WCR inhibited the production of IL-23 for 1.8-fold, 1.3-fold, and 7-fold, respectively, compared with the HaCaT cells stimulated with TNF-*α* and IFN-*γ* alone (Figure 4(c)).

## 4. Discussion

Traditional herbal medicines are clearly attractive drug candidates in health maintenance for Asian people and becoming more frequently used in the western countries [17]. Only one system of folk remedy officially accepted by Thai Ministry of Public Health is a traditional herbal medicine based on the element theory, consisting of earth (solidity), water (fluidity), fire (heat), and wind (motion). Psoriasis is caused by an imbalance among these four elements [18]. WCR is composed of 8 different crude plant extracts and has been shown to be effective for alleviating psoriasis of patients at Thai Traditional Medicine Clinic of Prapokklao Hospital since 2006. A previous study has shown that WCR has a clinical effect of 93% on blood-heat type psoriasis [11]. However, preclinical evidence defining how WCR works is still lacking. The present study investigated a specific mechanism underlying the therapeutic potential of WCR to be certain whether WCR has antiproliferation and anti-inflammatory effects.

As a primary bioassay screening of the potential drugs for psoriasis treatment, the inhibition of keratinocyte proliferation is considered as a crucial parameter. At the tested concentrations of 0–1000 *μ*g/mL, WCR had cytotoxicity effect on HaCaT cells as measured by SRB assay. Consistent with the SRB assay result, the cell viability was examined by CCK-8 assay, which determined mitochondrial dehydrogenase activities in the living cells [19]. WCR had a significant cytotoxicity effect on HaCaT cells in a concentration-dependent manner, with IC_50_ approximately 600 *μ*g/mL. The results strongly suggest that WCR contains active constituents that suppress growth of the cell and stimulate cell death. Unsurprisingly, acitretin and methotrexate, which are the drug of choice for the conventional psoriasis treatment, exhibited strong inhibitory effect on the cells. Previous studies have been reported about the effect of* S. glabra* on HaCaT cell lines, showing IC_50_ more than 200 *μ*g/mL [20]. It is therefore reasonable that* S. glabra* may be considered to be a major active ingredient of WCR that plays a crucial role in inducing cytotoxicity. In the present study, exposure of cells with WCR at concentrations over 100 *μ*g/mL resulted in dramatic inhibition of HaCaT cell growth, whereas treating HaCaT cells for 24 h with less than 100 *μ*g/mL WCR resulted in a 90% survival rate. Although these concentrations of WCR do not kill cells, they may play significant roles in suppressing some important signal transductions such as cell cycle and inflammation pathways.

Cell cycle analysis was performed to elucidate whether WCR interferes with a normal cycle of cell division. Therefore, three nontoxic concentrations of WCR (25, 50, and 75 *μ*g/mL) were examined for its effect on each phase of cell cycle compared with the positive controls, including methotrexate and acitretin. The results indicated that WCR and acitretin had no effect on the induction of cell cycle arrest, while methotrexate significantly induced S-phase arrest. In line with these results, previous evidence demonstrated that methotrexate acts specifically on the process of DNA synthesis (S-phase) by means of its irreversibly binding to the enzyme dihydrofolate reductase [21, 22]. The binding of methotrexate to dihydrofolate reductase results in a depletion of intracellular tetrahydrofolate pools and leads to a reduction of DNA synthesis and, therefore, exerts its cytotoxic activity in a cell-cycle-specific manner [21, 22]. The methotrexate toxicity, ranging from the unpleasant to the fatal, has severely limited its dose and duration of treatment [21, 22]. Moreover, in accordance with Lin et al., acitretin did not affect the viability of normal keratinocytes, but it was able to induce apoptosis in skin squamous cell carcinoma (SCL-1) cells [23]. In contrast, Liwei et al. revealed that retinoids altered cell cycle distribution of tongue squamous cell carcinoma (Tca8113) cells by increasing G0/G-phase population, decreasing in S-phase population, and inhibiting of G1/S switching [24]. In the present study, WCR did not cause any difference in the cell cycle profile, indicating that particular concentrations of WCR do not interfere with normal function of molecular executors in cell cycle signaling and, therefore, do not interrupt the cell cycle.

Extensive evidence has shown the involvement and role of T cells with a mixed proinflammatory Th1/Th17 cytokine in pathogenesis of psoriasis through causing inflammation, neovascularization, and hyperproliferation of keratinocytes [25, 26]. Herein, we found that HaCaT cells treated with TNF-*α* and IFN-*γ* alone markedly decreased the expression of IL-10 but increased that of various cytokine genes, including IL-1*β*, IL-6, IL-8, IL-17A, IL-22, IL-23, and TNF-*α* in HaCaT cells. This finding is also consistent with that of previous studies [27–29]. In contrast, the level of IL-10 gene expression after TNF-*α* and IFN-*γ* exposure was significantly potentiated by both 50 *μ*g/mL and 100 *μ*g/mL of WCR. It should be noted that IL-10 produced by regulatory T cells has been characterized as an anti-inflammatory cytokine and shown to suppress proinflammatory T cell production and keratinocyte inflammatory markers [26, 30]. As IL-10 has demonstrated promise in a phase II trial for cutaneous disorders [30], therefore, our observation that IL-10 gene was drastically upregulated suggests that WCR may have antipsoriatic properties.

WCR exerts anti-inflammatory activity since the extract at 50 *μ*g/mL and 100 *μ*g/mL significant decreases the expression of various inflammatory cytokines (IL-1*β*, IL-6, IL-8, IL-17A, IL-22, IL-23, and TNF-*α*) in HaCaT cells stimulated with TNF-*α* and IFN-*γ*. In agreement with our findings, previous reports do suggest that terpenes and flavonoids found in* A. galanga*, which is one of major active components of WCR, have strong antioxidant and anti-inflammatory activities [31].* A. galanga* can also reduce the expression of NF-*κ*B signaling biomarkers via an increased expression of a molecule inhibiting NF-*κ*B (TNFAIP3) [10], leading to a decrease in cytokine production by T cells [31]. Other studies reported that the flavonoid astilbin isolated from the rhizome of* S. glabra*, another major active component of WCR, has an inhibitory effect on Jak/Stat3 signaling in Th17 cells [32] and inhibits production of nitric oxide, TNF-*α*, and IL-6 production [33] through p65, ERK1/2, and JNK pathways [34]. Astilbin also downregulates T cell function by suppressing activated T cell migration and adhesion and stimulating negative regulatory interleukin 10 (IL-10) production [35, 36]. In addition, anti-inflammatory, antiproliferative, and antioxidant activities have been well-documented for other components of WCR, including* S. corbularia* [37],* R. nasutus *[38, 39],* A. ebracteatus* [40],* S. involute*,* S. collinsae* [41], and* Smilax* sp. [42].

At the protein level, we found a large difference in the production of IL-17A, IL-22, and IL-23 between the WCR-treated and untreated groups. While only TNF-*α* and IFN-*γ* treated HaCaT cells secreted large amounts of IL-17A, IL-22, and IL-23, 20 *μ*M of acitretin and 100 *μ*g/mL of WCR significantly inhibited the TNF-*α*- and IFN-*γ*-stimulated IL-17A, IL-22, and IL-23 secretion. Although 50 *μ*g/mL of WCR did not show statistically significant reduction of IL-17A, IL-22, and IL-23 levels, it had the tendency to reduce the production of these inflammatory cytokines. Herein, the effect of WCR on inhibition of TNF-*α*- and IFN-*γ*-induced cytokine production highlighted a remarkable correlation between mRNA expression and protein secretion for IL-17A, IL-22, and IL-23 biomarkers. A previous study has demonstrated that myeloid cell-produced IL-23 stimulates the differentiation of Th17 cells, leading to expansion and survival of these T cells [43]. Once Th17 cells are activated, they produce many mediators, including IL-17A and IL-22 which consequently induce proliferation of keratinocytes and other clinical symptoms of psoriasis [3, 44]. Recently, the novel biologic agents that specifically target IL-17A and the p40 subunit of IL-12 and IL-23 have been used effectively in the treatment of psoriasis [45, 46]. Unfortunately, the limitations of these biologic drugs are ten-times higher direct costs than conventional systemic drugs [3], as well as other main concerns regarding the potential to increase infection and the risk of cancer from long-term chronic immunosuppression [6]. Thus, this study suggests that WCR, which is an affordable traditional medicine formulation, likely exerts antipsoriatic inflammation by inhibiting the expression of IL-17A, IL-22, and IL-23.

## 5. Conclusion

As part of our effort to develop an alternative treatment of antipsoriasis from natural products, the formulation of WCR crude extract at low concentrations was successfully proved to have potent anti-inflammatory effects, without having cytotoxicity or inducing cell cycle arrest, on the HaCaT cells. This indicates that WCR could be a promising candidate for further investigation and development for psoriasis therapy. To our knowledge, the current study is the first report to demonstrate the effect of WCR at the molecular level. This is a valuable information for developing reliable therapies. However, more accumulated evidence is needed to reveal all aspects of antipsoriatic properties of WCR to be ensured that WCR is effective and safe for use in psoriasis patients.

## Figures and Tables

**Table 1:** Plants used in WCR.

	Thai name	Scientific name	Family name	Part used	Original source
1	Kha	*Alpinia galanga* (L.) Willd.	Zingiberaceae	Rhizomes	Chanthaburi Province, Thailand
2	Khao Yen Tai	*Smilax glabra* Wall.ex Roxb.	Smilacaceae	Rhizomes	
3	Khao Yen Nuea	*Smilax corbularia* Kunth.	Smilacaceae	Rhizomes	
4	Khao Yen Jeen	*Smilax *sp.	Smilacaceae	Rhizomes	

5	Hua Ta Pead	*Stemona involuta* Inthachub.	Stemonaceae	Roots	Chachoengsao Province, Thailand
6	Non Tai Yak	*Stemona collinsae *Craib.	Stemonaceae	Roots	

7	Thong Pan Chang	*Rhinacanthus nasutus* (L.) Kurz.	Acanthaceae	Aerial part	Chanthaburi Province, Thailand
8	Ngueak plaamo	*Acanthus ilicifolius *L.	Acanthaceae	Aerial part	

**Table 2:** Primer sequences.

Gene name	Sequence
Forward GAPDH	ACCACAGTCCATGCCATCAC
Reverse GAPDH	TCCACCACCCTGTTGCTGTA

Forward IL-1*β*	AGCTCGCCAGTGAAATGATG
Reverse IL-1*β*	TGGTGGTCGGAGATTCGTAG

Forward IL-6	CCACTCACCTCTTCAGAACG
Reverse IL-6	CATCTTTGGAAGGTTCAGGTTG

Forward IL-8	GGTGCAGTTTTGCCAAGGAG
Reverse IL-8	TTCCTTGGGGTCCAGACAGA

Forward IL-10	TGTTCTTTGGGGAGCCAACA
Reverse IL-10	GGCTCCCTGGTTTCTCTTCC

Forward IL-17A	GCTGATGGGAACGTGGACTA
Reverse IL-17A	TAGGCCACATGGTGGACAATC

Forward IL-22	CCAGCCTTATATGCAGGAGG
Reverse IL-22	TTTCAGCTTTGCTCTGGTCA

Forward IL-23	AAACCAGAGACGCGCTGAA
Reverse IL-23	GCAGCAACAGCAGCATTACA

Forward TNF-*α*	TGGGATCATTGCCCTGTGAG
Reverse TNF-*α*	CCAGGTTTCGAAGTGGTGGT
